# Early participant-reported symptoms as predictors of adherence to anastrozole in the International Breast Cancer Intervention Studies II

**DOI:** 10.1093/annonc/mdx713

**Published:** 2017-11-06

**Authors:** I Sestak, S G Smith, A Howell, J F Forbes, J Cuzick

**Affiliations:** 1 Centre for Cancer Prevention, Wolfson Institute of Preventive Medicine, Queen Mary University of London, London, UK; 2 Leeds Institute of Health Science, University of Leeds, Leeds, UK; 3 Division of Cancer Sciences, University of Manchester, Manchester, UK;; 4 Department of Surgical Oncology, Calvary Mater Newcastle Hospital, Newcastle, Australia

**Keywords:** breast cancer, prevention, adherence, endocrine therapy

## Abstract

**Background:**

Anastrozole reduces breast cancer risk in women at high risk, but implementing preventive therapy in clinical practice is difficult. Here, we evaluate adherence to anastrozole in the International Breast Cancer Intervention Study (IBIS)-II prevention and ductal carcinoma *in situ* (DCIS) trials, and its association with early symptoms.

**Patients and methods:**

In the prevention trial, 3864 postmenopausal women were randomized to placebo versus anastrozole. A total of 2980 postmenopausal women with DCIS were randomized to tamoxifen versus anastrozole. Adherence to trial medication was calculated using the Kaplan–Meier method and all *P*-values were two-sided.

**Results:**

In the prevention trial, adherence was 65.8% [anastrozole (65.7%) versus placebo (65.9%); HR = 0.97 (0.87–1.09), *P *= 0.6]. Adherence was lower for those reporting arthralgia in the placebo group (*P *= 0.02) or gynecological symptoms in the anastrozole group (*P *= 0.003), compared with those not reporting these symptoms at 6 months. In the DCIS study, adherence was 66.7% [anastrozole (67.5%) versus tamoxifen (65.8%); HR = 1.06 (0.94–1.20), *P* =* *0.4]. Hot flashes were associated with greater adherence in the anastrozole arm (*P *=* *0.02). In both studies, symptoms were mostly mild or moderately severe, and adherence decreased with increasing severity for most symptoms. Drop-outs were highest in the first 1.5 years of therapy in both trials.

**Conclusions:**

In the IBIS-II prevention and DCIS trials, over two-thirds of women were adherent to therapy, with no differences by treatment groups. Participants who reported specific symptoms in the IBIS-II prevention trial had a small but significant effect on adherence, which strengthened as severity increased. Strategies to promote adherence should target the first year of preventive therapy.

Key Message

Preventive therapy for women at high risk of developing breast cancer can reduce disease burden. Implementing preventive therapy in routine clinical practice for high-risk women has been proved difficult due to several factors, including side-effects. Interventions should be targeted at women within the first 18 months of preventive therapy, as this is when medication withdrawal is most likely.

## Introduction

Breast cancer is the most frequent cancer among women worldwide, with an estimated 1.67 million new cancer cases diagnosed in 2012 [1] and hence the prevention of breast cancer is a recognized priority [2]. There have been increases in female breast cancer incidence rates since the 1970s, although rates appear to be stabilizing among younger women and in more economically developed countries [3, 4]. 

Preventive therapy for women at high risk of developing breast cancer or those with a diagnosis of ductal carcinoma *in situ* (DCIS) can reduce disease burden. Aromatase inhibitors (AIs) reduce breast cancer risk among high-risk postmenopausal women. Data from the International Breast Cancer Intervention Study II (IBIS-II) prevention trial show that women randomly assigned to receive anastrozole (1 mg/day) were over 50% less likely to be diagnosed with breast cancer compared with those taking a matching placebo [5]. A 65% relative risk reduction of invasive breast cancer was shown among women taking exemestane compared with placebo in the MAP.3 trial [6]. 

Two major AI prevention trials have been done among women with DCIS. In the IBIS-II DCIS trial, anastrozole was non-inferior to tamoxifen in reducing breast cancer recurrence [7]. The National Surgical Adjuvant Breast and Bowel Project (NSABP) B-35 trial showed statistically significant improvements in breast cancer-free interval among women taking anastrozole compared with tamoxifen [8]. Implementing preventive therapy in routine clinical practice for high-risk women is difficult due to reluctance among clinicians to prescribe the medication [9], low patient uptake [10–12], and sub-optimal adherence to therapy [10, 12, 13]. A major factor affecting implementation is side-effects [14, 15]. Patients are less willing to initiate preventive therapy if they perceive it to be linked with side-effects [16]. In the IBIS-I prevention trial, rates of adherence were lower among women reporting side-effects, but this finding was consistent in both tamoxifen and placebo groups [13]. Little is known about the acceptance of anastrozole in women at high risk of developing breast cancer.

Data from the IBIS-II prevention trial indicate women taking anastrozole are more likely to experience musculoskeletal events, vasomotor symptoms, and hypertension compared with those taking placebo [5]. In the IBIS-II DCIS trial, women taking anastrozole were more likely to experience fractures, musculoskeletal events, hypercholesterolemia, and strokes compared with women taking tamoxifen [7]. Large proportions of women reported arthralgia and hot flashes with both placebo [5] and tamoxifen [7], indicating that anastrozole may not be solely responsible for these patient reported side-effects. Here, we assess the association between participant-reported symptoms on adherence to anastrozole in the IBIS-II prevention and DCIS trials.

## Methods

### Participants

The IBIS-II prevention study is an international, randomized, double-blind, and placebo-controlled trial conducted in 18 countries [5]. Postmenopausal women (*n *=* *3864) aged 40–70 years were randomly assigned to either 1 mg anastrozole or matching placebo daily for 5 years. The trial is registered, number ISRCTN31488319. The IBIS-II DCIS study recruited 2980 postmenopausal women with locally excised estrogen receptor positive or progesterone positive DCIS. Women were randomized to receive 1 mg/day oral anastrozole or 20 mg/day oral tamoxifen for 5 years [7]. The trial is registered, number ISRCTN37546358. Details of patient cohorts and characteristics are provided in supplementary material, available at *Annals of Oncology* online.

### Adherence

Adherence was defined as the period between trial randomization date and the date of the final follow-up visit [17]. Adherence (full/deviation/holiday/stopped) and further details on non-adherence were recorded on each follow-up CRF at 6-monthly visits. Pre-defined rules for assessing adherence were developed and used by SS to review all CRFs (supplementary material, available at *Annals of Oncology* online). Women who self-reported medication cessation at a visit were classified as non-adherent. Each woman was assessed for persistent use of medication for at least 4.5 years (adherent) or stopping before 4.5 years (non-adherent). All women in the prevention and DCIS IBIS-II trials have finished 5 years of active treatment.

### Participant symptoms

Symptoms were assessed at each follow-up visit using pre-defined items for arthralgia (arthritis, arthrosis, or joint disorder), hot flashes/night sweats, vaginal discharge, irregular vaginal bleeding, eye diseases/cataracts, and osteoporosis/fractures. Vaginal discharge and irregular vaginal bleeding were grouped together as gynecological symptoms because they are similar. All symptoms were classified as mild, moderate, or severe as judged by the women. The most severe gynecological symptom was used when computing this item.

### Statistical analysis

Adherence to trial medication was calculated using the Kaplan–Meier method [18], both overall and by treatment group separately. Further details of statistical methods are provided in supplementary material, available at *Annals of Oncology* online.

## Results

### IBIS-II prevention study

Postmenopausal women (*n *=* *3864) were randomized to receive 1 mg/day anastrozole versus matching placebo. Women were excluded from the current analysis if they did not start their allocated medication (*n *=* *77) or if they were ineligible (*n *=* *24) (supplementary Figure S1, available at *Annals of Oncology* online). Hence, 3763 women (97.4%) were included in this analysis (1868 anastrozole versus 1895 placebo). For the analyses investigating associations between early reported symptoms and adherence, those who did not reach the 6 month visit were excluded (*n *=* *159). Baseline participant characteristics were balanced between treatment groups (supplementary Table S1, available at *Annals of Oncology* online).

**Figure 1.**
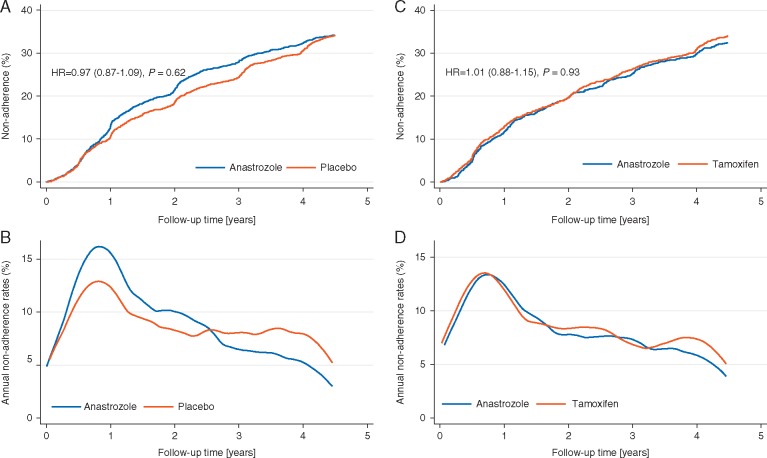
Kaplan–Meier plots for non-adherence and annual non-adherence rates (%) according to treatment arm for the IBIS-II prevention (A, B) and DCIS (C, D) studies. Kaplan–Meier curves were calculated and tested for equality using log-rank test. All statistical tests were two-sided. IBIS, International Breast cancer Intervention Study; HR, hazard ratio; CI, confidence interval.

Overall, 1287 women (34.2%) were non-adherent. For women randomized to anastrozole, adherence was non-significantly lower compared with those on placebo [HR = 0.97 (0.87–1.09), *P *=* *0.6] (Figure 1). Mean time on the treatment was similar in both treatment arms (anastrozole 3.90 years versus placebo 4.00 years). Overall, annual drop-out rates were highest within the first 12–18 months of follow-up (Figure 1) and declined sharply thereafter. The following predictors were significantly associated with adherence in the univariate model: 60 years or older, hysterectomy, oophorectomy, natural menopause, and previous participation in the IBIS-I prevention trial (supplementary Table S2, available at *Annals of Oncology* online). When adherence was investigated adjusted for all previous significant predictors, being older than 60 years of age [OR = 1.17 (1.01–1.34), *P *=* *0.03], not having had a hysterectomy [OR = 0.75 (0.59–0.96), *P *=* *0.03], and previous participation in the IBIS-I trial [OR = 1.38 (1.14–1.67), *P *=* *0.001] remained significant predictors for adherence.

At 6 months of follow-up (*n *=* *3604), significantly more women randomized to anastrozole compared with placebo reported arthralgia (31.5% versus 25.5%, *P *<* *0.001), hot flashes/night sweats (42.6% versus 34.1%, *P* < 0.001), and gynecological symptoms (11.4% versus 9.0%, *P *=* *0.02). Women reporting arthralgia [HR = 0.85 (0.75–0.97), *P *=* *0.01] or gynecological symptoms [HR = 0.78 (0.65–0.94), *P *=* *0.008] were significantly less likely to be adherent at 4.5 years than those not reporting these symptoms (Table 1). However, absolute differences in adherence were small.

**Table 1.** Early reported symptoms at 6 months associated with non-adherence in the IBIS-II prevention and DCIS study

IBIS-II prevention				IBIS-II DCIS			
	Non-adherence (%)	HR (95% CI)^a^	*P*-value		Non-adherence (%)	HR (95% CI)^b^	*P*-value
Arthralgia							
No (*n*=2577)	30.0	–	–	No (*n*=2069)	28.9	–	–
Yes (*n*=1027)	24.6	0.85 (0.75–0.97)	0.01	Yes (*n*=702)	31.2	0.90 (0.77–1.05)	0.2
Hot flashes/night sweats							
No (*n*=2224)	30.8	–	–	No (*n*=1561)	31.3	–	–
Yes (*n*=1380)	32.0	1.02 (0.90–1.15)	0.8	Yes (*n*=1210)	27.3	1.18 (1.02–1.36)	0.02
Gynecological							
No (*n*=3238)	30.6	–	–	No (*n*=2496)	29.4	–	–
Yes (*n*=366)	37.4	0.78 (0.65–0.94)	0.008	Yes (*n*=275)	30.9	0.93 (0.74–1.16)	0.5
Eye disease							
No (*n*=3424)	31.1	–	–	No (*n*=2657)	29.4	–	–
Yes (*n*=180)	34.4	0.90 (0.69–1.16)	0.4	Yes (*n*=114)	32.5	0.87 (0.63–1.22)	0.4
Osteoporosis							
No (*n*=3534)	31.3	–	–	No (*n*=2717)	29.5	–	–
Yes (*n*=70)	30.0	1.00 (0.65–1.55)	0.9	Yes (*n*=54)	31.5	0.92 (0.57–1.48)	0.7

a HR adjusted for age, hysterectomy, and previous IBIS-1 participation.

b HR adjusted for HRT.

IBIS, International Breast cancer Intervention Study; HR, hazard ratio; CI, confidence intervals.

**Figure 2.**
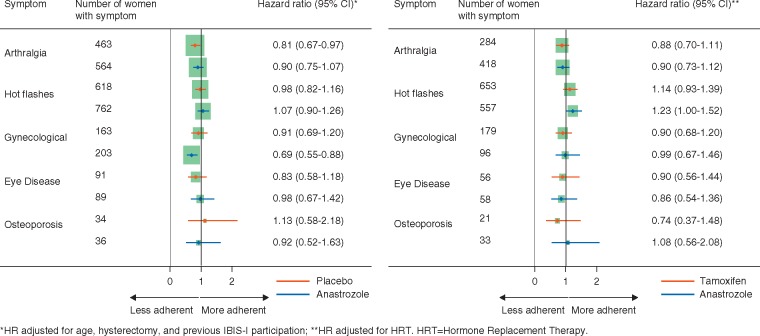
Forest plots for non-adherence (hazard ratios) among women reporting symptoms at 6 months by treatment arm for the IBIS-II prevention (A) and DCIS (B) studies. The squares represent the point estimates. Sizes of the squares represent the number of events. The horizontal error bars show the 95% confidence intervals (CI) of each hazard ratio. IBIS, International Breast cancer Intervention Study; CI, confidence interval.

For women reporting arthralgia at 6 months, only those randomized to placebo were significantly less adherent [HR = 0.81 (0.67–0.97), *P *=* *0.02]. There was no significant difference in non-adherence for those randomized to anastrozole [HR = 0.90 (0.75–1.07), *P *=* *0.2] (Figure 2). Women randomized to anastrozole and reporting gynecological symptoms were 31% less likely to be adherent at 4.5 years [HR = 0.69 (0.55–0.88), *P *=* *0.003] compared with those not reporting these symptoms (Figure 2). No difference in adherence was observed for those reporting gynecological symptoms in the placebo arm [HR = 0.91 (0.69–1.20), *P *=* *0.5]. For all other reported symptoms at 6 months, no significant differences between treatment arms were observed with regard to adherence (Figure 2). The majority of symptoms reported at 6 months among both treatment groups were of mild or moderate severity. Non-adherence was similar between those not reporting a symptom and those reporting mild symptoms at 6 months (supplementary Figure S2, available at *Annals of Oncology* online). We observed significant trends for non-adherence with increasing severity for all reported symptoms (*P *<* *0.001), except for eye diseases (*P *=* *0.8) and osteoporosis (*P *=* *0.9) (supplementary Figure S2, available at *Annals of Oncology* online).

### IBIS-II DCIS study

Postmenopausal women (*n *=* *2980) diagnosed with DCIS within 6 months before randomization were allocated to receive 1 mg/day anastrozole versus 20 mg/day tamoxifen. Women were excluded from the current analysis if they did not start the allocated medication (*n *=* *32) or if they were ineligible (*n *=* *18). This left 2930 women (98.3%) for the adherence analysis (1486 tamoxifen versus 1444 anastrozole) (supplementary Figure S1, available at *Annals of Oncology* online). For the analysis of the association of early reported symptoms and adherence an additional 159 women who did not reach the 6 month follow-up point were excluded (Figure 1). Baseline demographics were balanced between treatment groups (supplementary Table S1, available at *Annals of Oncology* online).

Overall, non-adherence was 33.3% and non-significantly different between anastrozole and tamoxifen [HR = 1.06 (0.94–1.20), *P *=* *0.4] (Figure 1). Mean time on study was also similar between treatment arms (anastrozole: 3.99 years versus tamoxifen: 3.95 years). As with the prevention study, rates of non-adherence were greatest within the first 12–18 months and decrease thereafter (Figure 1). In the univariate analysis, previous HRT use [OR = 0.79 (0.67–0.92), *P *=* *0.003], hysterectomy [OR = 0.83 (0.70–0.98), *P *=* *0.03], and oophorectomy [OR = 0.75 (0.58–0.97), *P *=* *0.03] were significantly associated with decreased likelihood of adherence. Women who had a natural menopause [OR = 1.25 (1.04–1.50), *P *=* *0.02] were significantly more likely to be adherent than their counterparts (supplementary Table S1, available at *Annals of Oncology* online). In the multivariate analysis, only previous HRT use remained a significant predictor of non-adherence [OR = 0.81 (0.69–0.95), *P *=* *0.009] in women with DCIS.

A total of 2770 women were included to investigate early reported symptoms and adherence. At 6 months, significantly more women randomized to anastrozole reported arthralgia compared with tamoxifen (30.4% versus 20.3%, *P *<* *0.001). In contrast, significantly more women randomized to tamoxifen reported hot flashes/night sweats (40.6% versus 46.7%, *P *=* *0.001) and gynecological symptoms (7.0% versus 12.8%, *P *<* *0.001). Women reporting hot flashes were significantly more adherent than those not reporting these symptoms [HR = 1.18 (1.02–1.36), *P *=* *0.02] (Table 1). Those on anastrozole reporting these symptoms were significantly more adherent than their counterparts [HR = 1.23 (1.00–1.52), *P *=* *0.05], and a non-significant increase in non-adherence was also observed for those on tamoxifen (Figure 2). For all other symptoms, no association with adherence was observed either overall or by treatment arm (Table 1 and Figure 2). Non-adherence was similar between those not reporting a symptom and those reporting mild symptoms at 6 months (supplementary Figure S2, available at *Annals of Oncology* online). We observed significant trends for non-adherence with increasing severity for arthralgia (*P *<* *0.001), hot flashes (*P *=* *0.009), and gynecological symptoms (*P *=* *0.03). There were no significant trends for any other symptoms (supplementary Figure S2, available at *Annals of Oncology* online).

## Discussion

In the IBIS-II prevention and DCIS trials, over one-third of women were non-adherent for the full course of therapy. There were no overall significant differences in study drop-outs by treatment arm for either study. Arthralgia and gynecological symptoms at any severity significantly reduced the likelihood of adherence in the placebo and anastrozole groups of the prevention trial, respectively. In the DCIS trial, hot flashes were associated with a higher likelihood of adherence. Associations between symptoms and non-adherence strengthened with increasing severity. Participant-reported early symptoms may be partially responsible for non-adherence to anastrozole preventive therapy, however, other factors are likely to play an important role.

Similarities in non-adherence between the treatment groups suggest that some factors associated with non-adherence are likely to be unrelated to anastrozole. Similar non-adherence rates between treatment arms were also reported in the MAP.3 prevention trial (32.8% exemestane versus 28.7% placebo) [19] and the NSABP-B35 trial (35.6% anastrozole versus 35.8% tamoxifen) [8]. Identifying strategies to reduce the burden of moderate and severe symptoms could be one approach increase medication adherence. However, identifying modifiable factors other than medication induced side-effects that explain non-adherence could improve behavioral interventions. Our previous systematic review of uptake and adherence to breast cancer chemoprevention suggests women’s perceived risk of breast cancer is likely to play a role [10].

Non-adherence in the IBIS-I tamoxifen prevention trial showed higher levels of study drop-outs in the first 12–18 months of therapy [13] and a finding has been reported in the adjuvant setting [20, 21]. We observed the same pattern in both the IBIS-II prevention and DCIS trials. This consistency highlights that this time period may be the most appropriate point to deliver interventions supporting adherence. Identifying the optimal timing of interventions is important, but there is a paucity of strategies shown to effectively improve adherence to endocrine therapy. Identifying modifiable determinants of adherence to preventive therapy should be prioritized, so they can be incorporated into strategies to improve medication taking behavior.

Participant-reported symptoms do not completely explain non-adherence to preventive therapy. Arthralgia, hot flashes/night sweats, and gynecological symptoms were more common among women taking anastrozole in the IBIS-II prevention trial. Fewer women taking anastrozole in the IBIS-II DCIS trial reported hot flashes/night sweats or gynecological symptoms, however arthralgia was more common compared with the tamoxifen treatment arm. The NSABP-B35 observed similar trends [22], but demonstrated no significant differences in quality of life between women taking anastrozole and tamoxifen. Strategies to manage these symptoms are required to ensure quality of life is not affected among women taking preventive therapy.

This study has strengths and limitations. We are among the first to provide a detailed report of the relationship between symptoms and non-adherence among women taking anastrozole for preventive therapy. The data derive from two large international randomized studies that were carefully monitored throughout. However, because these data were from motivated participants willing to enroll in a clinical trial, we may have over-estimated the proportion of women who are able to complete the full course of therapy. In addition, we were not able to investigate concurrent medication associated with symptoms relieve, which may contribute to better adherence. There is no gold standard measure of medication adherence, our reported outcome was recorded during clinic visits and may be an inflated estimate [23]. Quality of life assessments that encapsulate the full impact of symptoms on everyday life may be more closely associated with adherence [24]. We noted a weaker than expected relationships between symptoms reported at 6 months and non-adherence. One possibility is that late-onset symptoms could be responsible for subsequent trial drop-out. However, this is unlikely to account for a large amount of drop-outs as the majority of treatment induced side-effects occur in the first year of therapy [25]. Our 6-month assessment may also be identifying symptoms that are transient in nature. Women experiencing symptoms that persist for longer or become more severe may be more likely to drop-out, and this would not be captured by our analysis.

In conclusion, non-adherence with anastrozole was moderate in both the IBIS-II prevention and DCIS trials. Only women reporting moderate or severe symptoms were less likely to be adherent. Identifying factors other than medication induced side-effects that explain non-adherence could help to target future intervention strategies to support medication taking behavior. Once interventions have been developed, they should be targeted at women within the first 18 months of therapy, as this is when medication cessation is most likely.

## Supplementary Material

Supplementary Figure S1

Supplementary Figure S2

Supplementary Table S1

Supplementary Table S2

Supplementary Material
