# Supplementary figures and images for: Early participant-reported symptoms as predictors of adherence to anastrozole in the International Breast Cancer Intervention Studies II
Source: Ann Oncol. 2017 Nov 6;29(2):504–9. doi: 10.1093/annonc/mdx713 (PMC5834118; doi:10.1093/annonc/mdx713)

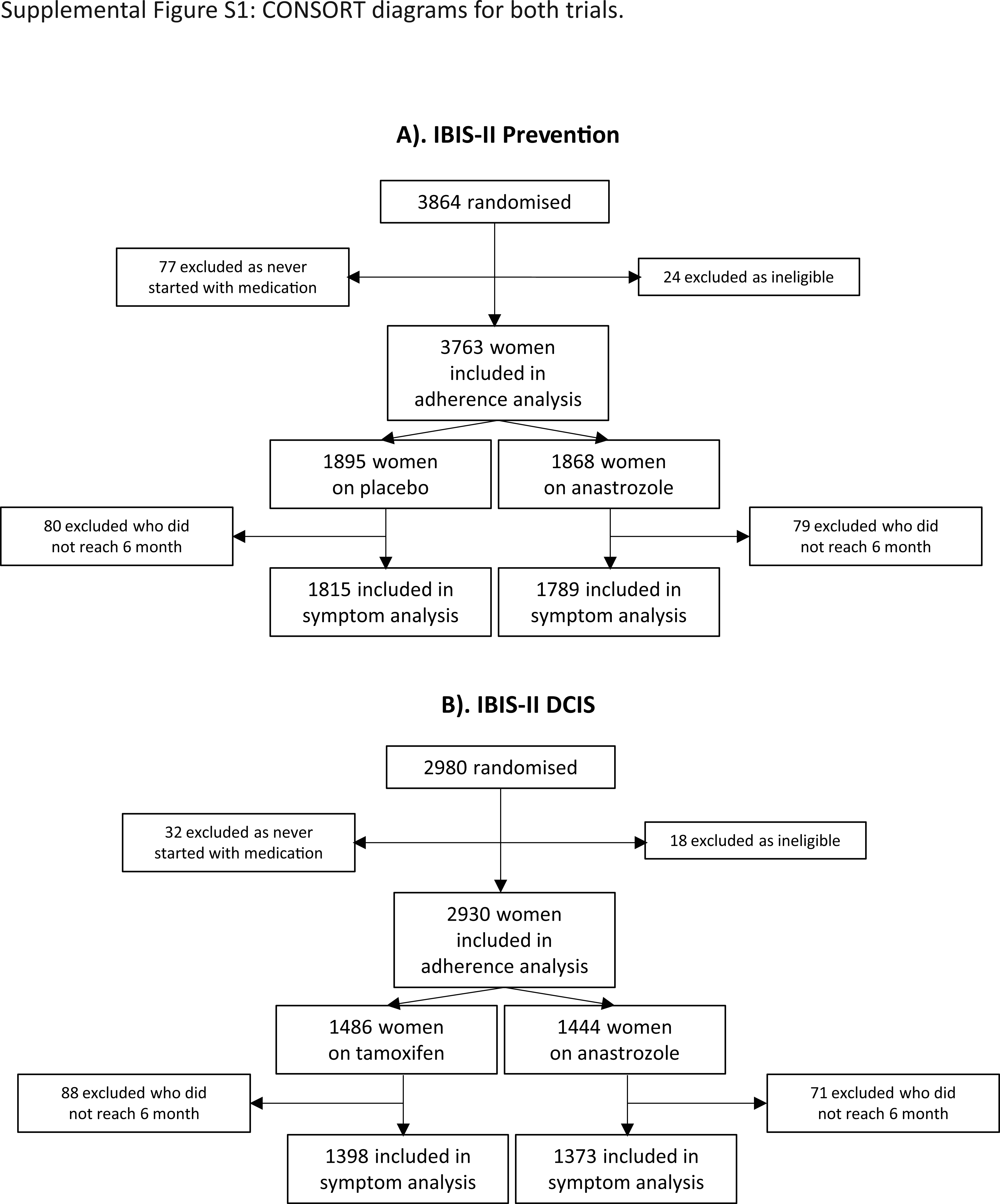

Supplement: Supplementary Figure S1 [file supplemental_figure_s1_mdx713.png]

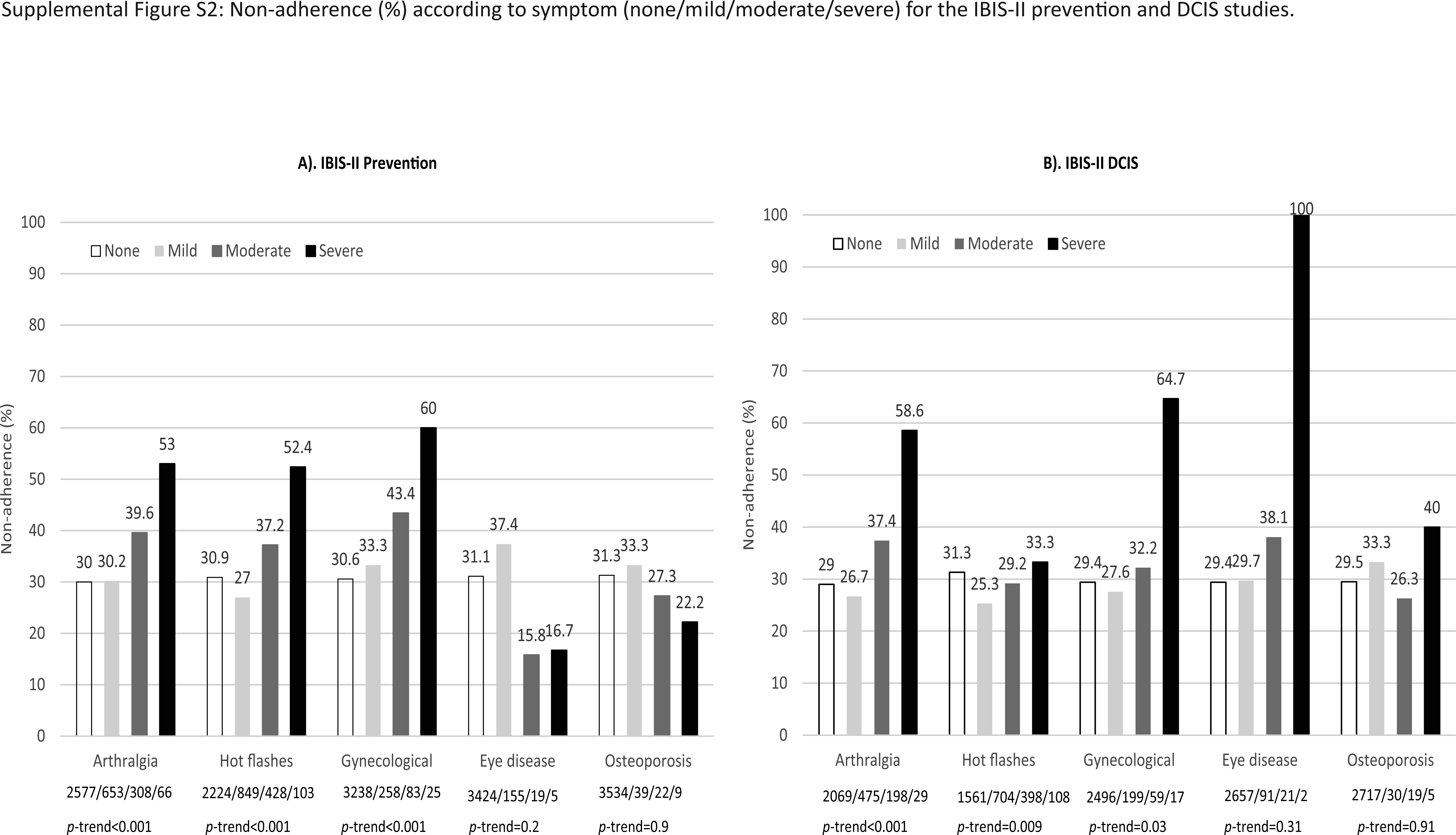

Supplement: Supplementary Figure S2 [file supplemental_figure_s2_mdx713.png]
