# Supplementary material for: Early participant-reported symptoms as predictors of adherence to anastrozole in the International Breast Cancer Intervention Studies II
Source: Ann Oncol. 2017 Nov 6;29(2):504–9. doi: 10.1093/annonc/mdx713 (PMC5834118; doi:10.1093/annonc/mdx713)
Supplement: Supplementary Table S1 [file supplemental_table_s1_mdx713.docx]

**Supplemental Table S1**: Baseline demographics for the IBIS-II prevention and DCIS study according to treatment arm.

|  | **IBIS-II Prevention** | | **IBIS-II DCIS** | |
| --- | --- | --- | --- | --- |
|  | **Placebo**  **(*n*=1895)** | **Anastrozole**  **(*n*=1868)** | **Tamoxifen**  **(*n*=1486)** | **Anastrozole**  **(*n*=1444)** |
| **Age (years), median (IQR)** | 59.4 (55.1-63.2) | 59.5 (55.0-63.6) | 60.3 (55.9-64.5) | 60.4 (56.4-64.7) |
| **BMI (kg/m^2^), median (IQR)** | 27.3 (24.4-31.2) | 27.5 (24.3-31.1) | 26.7 (23.7-30.1) | 26.7 (23.5-30.4) |
| **Nulliparous** | 273 (14.4%) | 269 (14.4%) | 232 (15.6%) | 238 (16.5%) |
| **Smoking status** |  |  |  |  |
| Never | 1112 (58.7%) | 1042 (55.8%) | 943 (63.4%) | 899 (62.3%) |
| Ex | 189 (10.0%) | 219 (11.7%) | 180 (12.1%) | 191 (13.2%) |
| Current | 579 (30.6%) | 589 (31.5%) | 312 (21.0%) | 307 (21.3%) |
| **Previous HRT use** | 897 (47.3%) | 884 (47.3%) | 653 (43.9%) | 669 (46.3%) |
| **Hysterectomy** | 644 (34.0%) | 625 (33.5%) | 404 (27.2%) | 406 (28.1%) |
| **Oophorectomy** | 275 (14.5%) | 246 (13.2%) | 143 (9.6%) | 132 (9.1%) |
| **IBIS-I** | 300 (15.8%) | 329 (17.6%) | - | - |

IBIS=International Breast cancer Intervention Study; IQR=Interquartile range; BMI=Body Mass Index; kg=kilogram; m=metre; HRT=Hormone Replacement Therapy.
