# Supplementary material for: Early participant-reported symptoms as predictors of adherence to anastrozole in the International Breast Cancer Intervention Studies II
Source: Ann Oncol. 2017 Nov 6;29(2):504–9. doi: 10.1093/annonc/mdx713 (PMC5834118; doi:10.1093/annonc/mdx713)
Supplement: Supplementary Table S2 [file supplemental_table_s2_mdx713.docx]

**Supplemental Table S2**: Factors associated with non-adherence in the univariate analysis in the IBIS-II prevention and DCIS study.

|  | **IBIS-II Prevention** | | |  | **IBIS-II DCIS** | | |
| --- | --- | --- | --- | --- | --- | --- | --- |
|  | **Non-adherence (%)** | **OR (95% CI)** | ***p*-value** |  | **Non-adherence (%)** | **OR (95% CI)** | ***p*-value** |
| **Age** |  |  |  | **Age** |  |  |  |
| ≤60 years (*n*=2002) | 36.0 | Reference | - | ≤60 years (*n*=1395) | 33.7 | Reference | - |
| >60 years (*n*=1761) | 32.1 | 1.19 (1.04-1.36) | 0.01 | >60 years (*n*=1535) | 33.0 | 1.03 (0.88-1.20) | 0.7 |
| **BMI (kg/m^2^)** |  |  |  | **BMI (kg/m^2^)** |  |  |  |
| <25 (*n*=1127) | 34.2 | Reference | - | <25 (*n*=1027) | 30.6 | Reference | - |
| 25-30 (*n*=1402) | 33.3 | 1.04 (0.88-1.23) | 0.7 | 25-30 (*n*=1024) | 34.3 | 0.84 (0.70-1.02) | 0.07 |
| >30 (*n*=1176) | 34.6 | 0.98 (0.83-1.16) | 0.8 | >30 (*n*=753) | 33.3 | 0.88 (0.72-1.08) | 0.2 |
| **Smoking status** |  |  |  | **Smoking status** |  |  |  |
| Never (*n*=2154) | 33.3 | Reference | - | Never (*n*=1842) | 31.2 | Reference | - |
| Ex (*n*=408) | 37.5 | 0.83 (0.67-1.04) | 0.1 | Ex (*n*=371) | 39.1 | 0.71 (0.56-0.89) | 0.003 |
| Current (*n*=1168) | 34.2 | 0.96 (0.93-1.12) | 0.6 | Current (*n*=619) | 33.3 | 0.92 (0.76-1.12) | 0.4 |
| **Previous HRT use** |  |  |  | **Previous HRT use** |  |  |  |
| No (*n*=1975) | 33.3 | Reference | - | No (*n*=1572) | 30.1 | Reference | - |
| Yes (*n*=1781) | 35.0 | 0.93 (0.81-1.06) | 0.3 | Yes (*n*=1322) | 35.4 | 0.79 (0.67-0.92) | 0.003 |
| **Previous OC use** |  |  |  | **Previous OC use** |  |  |  |
| No (*n*=846) | 35.0 | Reference | - | No (*n*=1054) | 31.0 | Reference | - |
| Yes (*n*=2851) | 33.5 | 1.07 (0.91-1.25) | 0.4 | Yes (*n*=1556) | 33.7 | 0.88 (0.75-1.04) | 0.2 |
| **Hysterectomy** |  |  |  | **Hysterectomy** |  |  |  |
| No (*n*=2487) | 31.5 | Reference | - | No (*n*=2083) | 31.4 | Reference | - |
| Yes (*n*=1269) | 39.2 | 0.71 (0.62-0.82) | <0.001 | Yes (*n*=810) | 35.6 | 0.83 (0.70-0.98) | 0.03 |
| **Oophorectomy** |  |  |  | **Oophorectomy** |  |  |  |
| No (*n*=3235) | 33.2 | Reference | - | No (*n*=2618) | 31.9 | Reference | - |
| Yes (*n*=521) | 39.3 | 0.77 (0.63-0.93) | 0.006 | Yes (*n*=275) | 38.5 | 0.75 (0.58-0.97) | 0.03 |
| **Nulliparous** |  |  |  | **Nulliparous** |  |  |  |
| No (*n*=3194) | 34.6 | Reference | - | No (*n*=2386) | 32.8 | Reference | - |
| Yes (*n*=542) | 30.6 | 1.20(0.98-1.46) | 0.07 | Yes (*n*=470) | 30.8 | 1.09 (0.88-1.35) | 0.4 |
| **Natural menopause** |  |  |  | **Natural menopause** |  |  |  |
| No (*n*=1088) | 39.5 | Reference | - | No (*n*=671) | 36.4 | Reference | - |
| Yes (*n*=2668) | 31.9 | 1.40 (1.21-1.62) | <0.001 | Yes (*n*=2223) | 31.4 | 1.25 (1.04-1.50) | 0.02 |
| **IBIS-I** |  |  |  |  |  |  |  |
| No (*n*=3134) | 35.3 | Reference | - |  |  |  |  |
| Yes (*n*=629) | 28.5 | 1.37 (1.14-1.66) | 0.001 |  |  |  |  |

IBIS=International Breast cancer Intervention Study; DCIS=Ductal Carcinoma In Situ; OR=Odds Ratio; CI=Confidence Interval; BMI=body mass index; kg=kilogram; m=metre; HRT= Hormone Replacement Therapy;

OC=Oral Contraceptive; IBIS=International Breast cancer Intervention Study.
