# Supplementary material for: Early participant-reported symptoms as predictors of adherence to anastrozole in the International Breast Cancer Intervention Studies II
Source: Ann Oncol. 2017 Nov 6;29(2):504–9. doi: 10.1093/annonc/mdx713 (PMC5834118; doi:10.1093/annonc/mdx713)
Supplement: Supplementary Material [file supplemental_material_mdx713.docx]

**Supplemental Material**

**Early participant-reported symptoms as predictors of adherence to anastrozole in the International Breast Cancer Intervention Studies II**

**Methods**

*Participants*

The IBIS II prevention study is an international, randomized, double-blind, placebo-controlled trial conducted in 18 countries [5]. Postmenopausal women (*n*=3864) aged 40-70 years were randomly assigned to either 1mg anastrozole or matching placebo daily for 5 years. The trial is registered, number ISRCTN31488319. The IBIS-II DCIS study recruited 2980 postmenopausal women with locally excised estrogen receptor positive or progesterone positive DCIS from 14 countries. Women were randomized to receive 1mg/day oral anastrozole (*n*=1471) or 20mg/day oral tamoxifen (*n*=1509) for 5 years [7]. Women were aged between 40-70 years, postmenopausal and had DCIS diagnosed within six months prior to randomisation. The trial is registered, number ISRCTN37546358. Full inclusion and exclusion criteria for both trials are available elsewhere [5, 7]. For this adherence analysis women were excluded if they did not commence with study therapy or were ineligible.

Participant characteristics were extracted from baseline case report forms (CRFs) including: age (≤60years, >60 years), body mass index (BMI) (<25, 25-30, >30, unknown), parity (yes/no), smoking status (never, current, ex), previous hormone replacement therapy (HRT) use (never, current, ex), hysterectomy (yes/no), natural menopause (yes/no), oophorectomy (yes/no) and natural menopause (yes/no). Previous participation in IBIS I was recorded for women in the prevention trial. All women were followed up by six monthly visits for the duration of the active treatment period (5 years). At each visit pre-defined symptom data were obtained from the CRFs and current use of assigned medication was recorded. Local ethics committees approved the trial at each participating institution.

*Adherence*

Adherence was defined as the period between trial randomization date and the date of the final follow-up visit [17]. Adherence (full/deviation/holiday/stopped) and further details on non-adherence were recorded on each follow-up CRF at six-monthly visits. Pre-defined rules for assessing adherence were developed and used by SS to review all CRFs (Online appendix). Women who self-reported medication cessation at a visit were classified as non-adherent. Each woman was assessed for persistent use of medication for at least 4.5 years (adherent) or stopping before 4.5 years (non-adherent). All women in the prevention and DCIS IBIS-II trials have finished 5 years of active treatment.

*Participant symptoms*

Symptoms were assessed at each follow-up visit using pre-defined items for arthralgia (arthritis, arthrosis, or joint disorder), hot flashes/night sweats, vaginal discharge, irregular vaginal bleeding, eye diseases/cataracts, and osteoporosis/fractures. Vaginal discharge and irregular vaginal bleeding were grouped together as gynecological symptoms because they are similar. All symptoms were classified as mild, moderate, or severe as judged by the women. The most severe gynecological symptom was used when computing this item.

*Statistical analysis*

Adherence to trial medication was calculated using the Kaplan-Meier method [18], both overall and by treatment group separately. Women who developed breast cancer or died within the 5 years of follow-up were censored at time of diagnosis or death. Baseline participant characteristics, treatment arm and the occurrence of symptoms within month 6 were analysed for the prediction of adherence using logistic regression. Significant predictors of adherence from the univariate analysis were included in the multivariate analyses. Cox regression models were used to assess adherence between early reported symptoms (at 6 months) and treatment allocation. The analyses testing the effect of symptom severity on adherence were based on comparisons of proportions. We used a non-parametric test of trend for the ranks of across ordered groups [19]. All *p*-values were two-sided and all confidence intervals (CIs) were at the 95% level. All calculations were performed using STATA version 13.1.

Rules to determine non-adherence:

- Definition = Time between initiation and cessation of trial medication is less than 4.5 years
  - Described additionally in notes as:
    - ‘Off study ‘ = not adherent
    - ‘Poor compliance’ = not adherent
    - ‘Cannot be contacted’ = not adherent
    - ‘Stopped for more than one month’ = non-adherent
    - ‘Took on alternate days for >6 months/ no timeframe given’ = non-adherent
  - If participant did not attend appointment, and did not attend any subsequent appointments = non adherent
